# ﻿Aquatic beetle diversity from Volcán Tacaná, Mexico: altitudinal distribution pattern and biogeographical affinity of the fauna

**DOI:** 10.3897/zookeys.1111.68665

**Published:** 2022-07-11

**Authors:** Alba Magali Luna-Luna, Caleb Califre Martins, Andrés López-Pérez, Andrés Ramírez-Ponce, Atilano Contreras-Ramos

**Affiliations:** 1 Doctorado en Ciencias Biológicas y de la Salud, Universidad Autónoma Metropolitana, Mexico City, Mexico; 2 Postdoctoral fellow, Instituto de Biología, Departamento de Zoología, Universidad Nacional Autónoma de México, Mexico City, Mexico; 3 Laboratorio de Ecosistemas Costeros, Departamento de Hidrobiología, Universidad Autónoma Metropolitana-Iztapalapa, Mexico City, Mexico; 4 Red de Biodiversidad y Sistemática, Instituto de Ecología, A. C., Xalapa, Veracruz, Mexico; 5 Instituto de Biología, Departamento de Zoología, Universidad Nacional Autónoma de México, Mexico City, Mexico

**Keywords:** Aquatic Coleoptera, Central American Nucleus, Chiapas, faunistics, PAE

## Abstract

Results of an aquatic beetle survey at Volcán Tacaná, Mexico, are presented with five altitudinal levels in a monthly sampling regime, aiming to estimate both diversity and altitudinal distribution patterns of the aquatic beetle fauna. The first list of aquatic beetle species from this mountain is presented, comprising 40 species in 32 genera and nine families, with four species recorded for the first time from Mexico and six recorded for the first time from Chiapas. The aquatic beetle fauna is characterized by Elmidae with 20 species, Dytiscidae with eleven species, Dryopidae with three, and Epimetopidae, Hydraenidae, Hydrophilidae, Gyrinidae, Lutrochidae, and Noteridae with one species each. The species composition through the sampled altitudinal gradient (670–1,776 m) was not homogeneous, with the elmid genera *Macrelmis*, *Heterelmis*, *Microcylloepus*, and *Austrolimnius* present at all levels, while *Hexanchorus*, *Neoelmis*, and *Onychelmis* were present at levels 1–3 (673–1,214 m); dytiscids were mostly present at levels 4 and 5 (1,619–1,776 m), and dryopids were present only at levels 1–3. A Parsimony Analysis of Endemicity supports a general partition between altitudinal levels 1–3 and levels 4 + 5.

## ﻿Introduction

Among the aquatic insects, aquatic beetles (Coleoptera) are one of the largest groups, with ca. 13,000 described species distributed in 30 families in three of the four coleopteran suborders (Short 2017). Within this insect group, the families Dytiscidae and Hydrophilidae are the largest, with ca. 4,300 and 2,900 species, respectively (Szczepański et al. 2018; Nilsson and Hákej 2020). Aquatic beetles are considered to have a great potential for biodiversity and conservation assessment of water habitats, besides their use as water quality indicators (Samir 2017). They have been recorded in all continents, except Antarctica, and inhabit almost all kinds of aquatic habitats from the smallest phytotelmata to large lakes and rivers (Bilton et al. 2019). Their distribution is determined by different ecological factors, including altitude, which plays an important role in aquatic beetle assemblage composition (Pérez-Bilbao et al. 2014).

Previous studies in the Neotropics have found that altitude may have a significant influence on the composition and structure of an aquatic insect community, as some genera may show a wide range of distribution, while others are characteristic of a particular altitudinal level (e.g., Arias 2004; Henriques-Oliveira and Nessimian 2010, in Brazil; González-Córdoba et al. 2015, 2016, 2020; Mosquera-Murillo and Sánchez-Vázquez 2018, in Colombia; Huanachin-Quispe and Huamantico-Araujo 2018, in Peru).

Approximately 583 species of aquatic Coleoptera are known from Mexico (Santiago-Fragoso and Spangler 1995; Arce-Pérez and Roughley 1999), although the actual number is probably greater. Several studies about local aquatic beetle diversity have taken place in Mexico, often aimed to evaluate the ecological condition of riparian systems, yet providing information on a still fragmentary view of this group’s biodiversity (e.g., Arce-Pérez and Novelo-Gutiérrez 1990, 1991, 2015; Arce-Pérez 1995; Arce-Pérez and Roughley 1999; Santiago-Fragoso and Sandoval-Manrique 2001; Arce-Pérez et al. 2002; Gómez-Anaya et al. 2004; Navarrete-Heredia and Zaragoza-Caballero 2006; Campbell et al. 2008; Arce-Pérez and Morón 2011; Torres-García and Pérez-Munguía 2013).

The Tacaná volcano, in the southern Mexican state of Chiapas and bordering Guatemala, is a key element of Volcán Tacaná Biosphere Reserve, a protected area relevant for its rich biotic, cultural, and economic value. This reserve is at the northernmost range of the Central American Nucleus or Central American Volcanic Arc and lies within the Mesoamerican Biological Corridor (CONANP 2013), a dynamic biogeographical area resulting from the assembly of biotas of Nearctic and Neotropical origin. Understanding the geographical distribution and the local diversity of aquatic insects is important to assess the patterns and processes of biological diversification (Benzina et al. 2019). This study aims to record the aquatic beetle diversity from Volcán Tacaná as well as to assess their altitudinal distribution patterns and the biogeographic affinities of the fauna to aid our understanding of biological diversification in the region.

Aquatic entomology, taxonomy, biodiversity, and tropical ecosystems might be a few defining keywords in Ralph Holzenthal’s philosophy as an academic advisor. These are relevant themes of encouragement for descriptive taxonomy and biodiversity exploration through several years of competing fields of knowledge, such as morphological and molecular approaches to systematics, which in the end are sides of the same disciplinary coin. This contribution is proudly dedicated by ACR, after 25 years of graduation, to Ralph’s bright academic career, in the company of young colleagues and AMLL, currently a graduate student and future academic grandchild.

## ﻿Materials and methods

### ﻿Study area

The Tacaná volcano, with its summit at 4,092 m asl, is located in southeastern Chiapas state, Mexico, 30 km NE of Tapachula, with its NE half lying in Guatemala. It is part of the Sierra Madre de Chiapas and lies within the Volcán Tacaná Biosphere Reserve, recognized by UNESCO since 2006. This reserve is located in the Chiapas coast hydrological region (RH-23), on the Pacific slope, and includes the basins of the Suchiate, Coatán, Cahoacán, and Cosalapa rivers (CONANP 2013). The reserve exhibits the following climates: humid temperate (higher portions of the volcano at ≥ 2,000 m; mean annual T = 15.3 °C), humid semi-warm (mid portions of the volcano ca. 1,300–2,000 m; mean annual T = 20.7 °C), and humid warm (lower portions of the volcano at ≤ 1,300 m; mean annual T = 24.3 °C), all with abundant summer rains (mean annual rainfall = 4,438.28 mm).

### ﻿Sampling procedures

**Figure 1.**
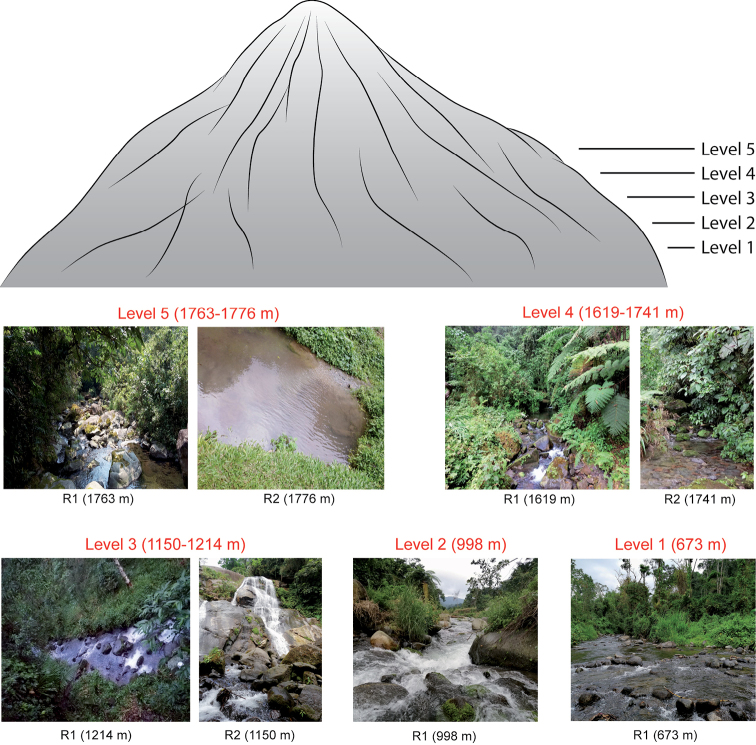
Levels and sampling sites for the aquatic beetle survey along an altitudinal gradient at Volcán Tacaná, Chiapas, Mexico, with habitat examples.

**Figure 2.**
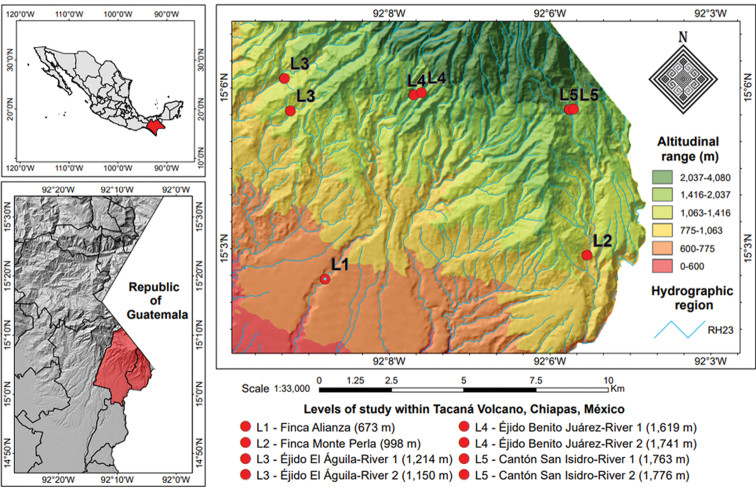
Distribution map of the five sampling levels and for the aquatic beetle survey along an altitudinal gradient at Volcán Tacaná, Chiapas, Mexico.

Five sampling localities were established, each at an altitude level along the volcano (levels 1–5; Figs 1, 2; Table 1), in order to estimate an altitudinal distribution pattern of species. Besides single sampling sites at each level (locality), levels 3–5 each had a second sampling site (i.e., there was a total of eight sampling sites; Fig. 1, Table 1). Water body and level selection essentially followed availability of lotic systems, as lentic systems are generally missing except for a crater lake at the top of the volcano; absence of permanent streams at higher elevations precluded sampling at uniformly separated levels, particularly between levels 4 and 5.

**Table 1.** Distribution of aquatic beetle species (Coleoptera) in the sampling levels and sites of Volcán Tacaná, Chiapas, Mexico. 0 = absent; 1 = present. Nea = Neartic; Neo = Neotropical.

FAMILY	SPECIES	SAMPLING POINTS								BIOG. REGIONS
		Lv1	Lv2	Lv3		Lv4		Lv5		
		R1	R1	R1	R2	R1	R2	R1	R2	
**Dryopidae**	1.*Dryopsmexicanus*	1	1	1	1	0	0	0	0	Neo.
	2.*Elmoparnuspandus*	1	0	0	0	0	0	0	0	Neo.
	3.*Helichussuturalis*	1	1	1	1	0	0	0	0	Nea.; Neo.
**Dytiscidae**	4.*Bidessonotuschampioni*	0	0	0	0	0	1	0	0	Neo.
	5.*Clarkhydrus* sp.	0	0	0	0	1	1	1	0	Neo
	6.*Copelatusdistinctus*	0	0	1	1	1	1	1	1	Nea.; Neo.
	7.*Ilybiosomaflohrianum*	0	0	0	0	0	1	0	1	Neo.
	8.*Laccophilusproximus*	0	0	0	0	1	0	1	0	Nea.; Neo.
	9.*Liodessusaffinis*	0	0	0	0	1	0	0	0	Nea.; Neo.
	10.*Neoclypeodytesfryii*	0	0	0	0	1	1	0	0	Nea.; Neo.
	11.*Platambusamericanus*	0	0	0	0	1	1	1	1	Neo.
	12.*Rhantusgutticollis*	0	0	0	0	0	0	0	1	Nea.; Neo.
	13.*Thermonectusnigrofasciatus*	0	0	0	0	0	0	0	1	Nea.; Neo.
	14.*Uvarussubornatus*	0	0	0	0	0	1	0	0	Neo.
**Elmidae**	15.*Austrolimniusformosus*	1	1	1	1	1	1	1	1	Neo.
	16.*Austrolimniussulcicollis*	1	1	1	1	1	1	1	1	Neo.
	17.*Cylloepusatys*	1	1	1	0	0	1	1	1	Neo.
	18.*Heterelmisglabra*	1	1	1	1	1	1	1	1	Nea.; Neo.
	19.*Heterelmisobesa*	1	1	1	1	1	1	1	1	Nea.; Neo.
	20.*Heterelmisobscura*	1	1	1	1	1	1	1	1	Nea.; Neo.l
	21.*Heterelmissimplex*	1	1	1	0	0	1	1	1	Neo.
	22.*Hexacylloepusmetapa*	1	1	1	1	0	1	1	1	Neo.
	23.*Hexanchorususitatus*	1	1	1	1	0	0	0	0	Neo.
	24.*Huleechiusspinipes*	1	1	1	1	0	1	1	1	Nea.; Neo.
	25.*Macrelmisgraniger*	1	1	1	1	1	1	1	1	Neo.
	26.*Macrelmisleonilae*	1	1	1	1	1	1	1	1	Neo.
	27.*Macrelmis* sp.	0	0	0	0	0	0	0	1	Neo.
	28.*Microcylloepusinaequalis*	1	1	1	1	1	1	1	1	Neo.
	29.*Microcylloepustroilus*	1	1	1	1	0	1	0	1	Neo.
	30.*Microcylloepus* sp.	1	1	1	1	1	1	1	1	Neo.
	31.*Neoelmisapicalis*	1	1	1	1	0	0	0	0	Nea.; Neo.
	32.*Onychelmislongicollis*	1	1	1	1	0	0	0	0	Neo.
	33.*Phanocerusclavicornis*	1	0	1	1	1	1	0	0	Nea.; Neo.
	34.*Xenelmisbufo*	1	1	1	1	1	1	1	1	Neo.
**Epimetopidae**	35.*Epimetopusthermarum*	1	0	0	0	0	0	0	0	Nea.; Neo.
**Gyrinidae**	36.*Gyretesboucardi*	1	0	0	0	0	0	0	0	Neo.
**Hydraenidae**	37.*Hydraena* sp.	1	1	1	1	1	1	1	1	Neo.
**Hydrophilidae**	38.*Tropisternusfuscitarsis*	0	1	0	0	0	0	0	0	Nea.; Neo.
**Luthrochidae**	39.*Lutrochus* sp.	1	0	0	0	0	0	0	0	Neo.
**Noteridae**	40.*Notomicrussharpi*	0	0	0	0	0	1	0	0	Nea.; Neo.

Level 1. Finca Alianza, municipality of Cacahoatán. The vegetation is evergreen tropical forest. The Cahoacán river (R1) belongs to the Cahoacán basin. The sampling site (15°02.429'N, 92°10.199'W) is located at 673 m asl.

Level 2. Finca Monte Perla, municipality of Unión Juárez. The vegetation is cloud forest. The river Cascada Monte Perla (R1) belongs to the Suchiate basin. The sampling site (15°02.870'N, 92°05.305'W) is located at 998 m asl.

Level 3. Ejido El Águila, municipality of Cacahoatán. The vegetation is cloud forest. On this locality, two rivers were sampled. The first river, La Resbaladilla (R1), belongs to the Cahotán basin, and the sampling site (15°05.564'N, 92°10.849'W) is at 1,214 m asl. The second river, Cascada La Sirena (R2), belongs to the Coatán basin, and the sampling site (15°06.160'N, 92°11.001'W) is located at 1,150 m asl.

Level 4. Ejido Benito Juárez El Plan, municipality of Cacahoatán. The vegetation is cloud forest. On this locality, two rivers were sampled, both belong to the Cahoacán basin. The first river, El Arroyo (R1), has its sampling site (15°05.946'N, 92°08.540'W) at 1,619 m asl. The second river, La Cascada (R2), has its sampling site (15°05.911'N, 92°08.396'W) at 1,741 m asl.

Level 5. Cantón San Isidro, municipality of Unión Juárez. The vegetation is pine forest. Two rivers were surveyed, both belong to the Suchiate basin. The first river (R1) has its sampling site (15°05.611'N, 92° 05.644'W) at an altitude of 1,763 m asl. The second river (R2) has its sampling site (15°05.588'N, 92°05.537'W) at 1,776 m asl.

The aquatic beetles were sampled monthly over a year (February 2018–February 2019). In each water body (sampling site) three points were selected, separated by 30 m from each other. Samples were obtained using a D-type benthos net (500 µm mesh), with a dimension of 30.5 cm wide × 53.3 cm long). A second trapping technique, a bucket black-light trap, was used for 3 hours at each sampling site. Captured specimens with organic matter surplus were stored in zippered plastic bags with 80% ethyl alcohol, which was replaced with clean alcohol after 24 hours; aquatic beetles were then sorted from other insect groups in the laboratory using a dissecting microscope, and subsequently identified.

### ﻿Taxonomic identification

The aquatic beetle specimens were dissected and identified to species using features of the genitalic structures; individual genitalia were extracted and stored in microvials with glycerin. Specimens were mounted on entomological pins, together with their associated labels and genitalia; specimens smaller than 12 mm were placed in paper cartons (points).

Identification was performed through introductory genus-level keys (White and Roughley 2008; Archangelsky et al. 2009; Miller and Bergsten 2016; Benetti et al. 2018; Passos et al. 2018), and subsequently with specialized revisions and original species descriptions.

All the material examined was deposited in the Colección Nacional de Insectos (CNIN) of the Instituto de Biología, Universidad Nacional Autónoma de Mexico.

### ﻿Parsimony Analysis of Endemicity (PAE)

To aid unravel a general distribution pattern of the aquatic beetle fauna along the altitudinal gradient in the volcano, we performed a Parsimony Analysis of Endemicity (**PAE**). According to Morrone (2009) “…PAE constructs cladograms based on the cladistic analysis of presence-absence data matrices of species and supraspecific taxa”. A matrix was built with distributional units (i.e., sampling sites) used as “terminals” and species serving as “characters”, aiming to obtain a hierarchical structure in the resulting most parsimonious cladograms. Because PAE has been applied to discern a biogeographical signal, such as delimiting areas of endemism or historical relationship between preexisting areas of endemism (Crisci et al. 2003), our assumption is that even a general pattern between altitudinal levels may be informative of a faunistic differentiation along the gradient.

Two analyses were applied: one with the main five levels of sampling (localities) as terminals (i.e., levels 3–5 had sites fused in a single unit), and a second with all eight sampling sites as distribution units or terminals (Table 1). Aquatic beetle species were used as characters, codified as present (1) or absent (0) at each of the distributional units (sampling sites or terminals). A hypothetical distributional unit with all species absent (zero vector) was used to root the trees.

The matrices (Table 1) were built with WinClada (Nixon 2002), then exported as a Nexus file to perform a parsimony analysis in TNT (Tree Analysis using New Technology, version 1.5) (Goloboff and Catalano 2016). The most parsimonious cladogram was obtained through a heuristic algorithm with parameters: random seed = 0, hold = 3000, hold / = 50 in a TBR (tree bisection and reconnection technique) of 60 replicates. The most parsimonious topology was exported to Adobe Illustrator CS5 software to be edited.

### ﻿Distribution maps

Mapping of the study site with the sampling sites was done with ArcGIS version 10.2. 2. Layers of states and municipalities were obtained from the National Institute of Statistics and Geography (**>INEGI**), with information on a 1:50,000 scale. Projection of localities with geographical coordinates was carried out with Universal Transverse Mercator (**UTM**). The raster of the CEM model of the Chiapas area was obtained, a cut of municipalities within the study area was made, with the help of a vector layer of municipal boundaries. The elevation model was adjusted with a reclassification of the z (altitude) values so altitude differences within our area of interest could be visualized. Seven intervals from 0 m to 4080 m were used for the reclassification. In addition, a shadow map (hillshade) was made to better visualize slopes of the terrain of the study area. Finally, layers of the watersheds are located on a scale of 1:50,000, which belongs to the Costa de Chiapas hydrographic region (key RH23).

### ﻿Results

In total, 23,295 specimens of aquatic beetles of 40 species, distributed in 32 genera and nine families (Dryopidae, Dytiscidae, Elmidae, Epimetopidae, Hydraenidae, Hydrophilidae, Gyrinidae, Lutrochidae, and Noteridae), were collected (Appendix 1). Elmidae had the highest richness with 20 species (50% of total richness). The lowest richness was recorded in Epimetopidae, Hydraenidae, Hydrophilidae, Gyrinidae, Lutrochidae, and Noteridae, with only one species each (2.5% richness, respectively).

We record the following four species from Mexico for the first time (Appendix 1): the Elmidae*Cylloepusatys* Hinton, 1946, *Hexacylloepusmetapa* Silva-Polizei, Barclay & Bispo, 2020, *Hexanchorususitatus* Spangler & Santiago-Fragoso, 1992, and the Dytiscidae*Bidessonotuschampioni* J. Balfour-Browne, 1947. Additionally, four species of Dytiscidae, *Ilybiosomaflohrianum* Sharp, 1887, *Liodessusaffinis* Say, 1823, *Neoclypeodytesfriyii* Clark, 1862, *Platambusamericanus* (Aubé, 1838), one species of Elmidae, *Huleechiusspinipes* (Hinton, 1934), and one of Noteridae, *Notomicrussharpi* J. Balfour-Browne, 1939, were recorded for the first time from the state of Chiapas.

### ﻿List of species of aquatic beetles (Coleoptera) from Volcán Tacaná, Mexico

Entries are arranged alphabetically by family and genus. Entries for genera include comments on number of species, and distribution. Species entries include the valid combination, distributional and altitudinal information, as well as type of substrate where they were collected. Altitude or elevation data are given in m above sea level.

## ﻿Results

### ﻿Family Dryopidae Billberg, 1820

#### ﻿Genus Dryops

Taxon classificationAnimaliaColeopteraDryopidae

Olivier, 1791

11A82A5C-2A9A-55A3-9C9C-05C0BD55A0F1

Dryops mexicanus Sharp, 1882

##### Note.

*Dryops* has a worldwide distribution and comprises 79 species (Shepard and Sites 2016), three of them are recorded from Mexico.

##### Distribution.

Mexico (Chiapas, Morelos), Belize, Costa Rica (Burgos and Trejo-Loyo 2001; Shepard 2004; Barr and Shepard 2017; Zaragoza-Caballero et al. 2019). It has been recorded at an altitudinal range of 200 to 840 m (Barr and Shepard 2017); in this study *D.mexicanus* was collected at levels 1 (670 m), 2 (934 m), and 3 (1,126–1,194 m).

##### Comments.

Collected on substrates consisting of gravel, macrophytes, and leaf packs; found in all sampling months (February 2018 through February 2019, dry and rainy seasons); also collected with a bucket light trap.

#### ﻿Genus Elmoparnus

Taxon classificationAnimaliaColeopteraDryopidae

Sharp, 1882

196D50DC-20B2-5BBD-8D11-20F3CC400DFD

##### Note.

This genus includes eight species recorded in the Neotropics (Kodada and Jäch 2005), two of them are recorded in Mexico.

#### ﻿ Elmoparnus pandus

Taxon classificationAnimaliaColeopteraDryopidae

Spangler & Perkins, 1977

3D35F7AB-EF6A-53D9-A381-A8DCA0D49E54

##### Distribution.

Mexico (Chiapas, Oaxaca), Belize, Guatemala, Honduras, Costa Rica, Panama (Spangler and Perkins 1977; Barr and Shepard 2017). The known altitudinal range of this species is 200 to 1,219 m (Spangler and Perkins 1977; Barr and Shepard 2017). In this study, it was collected at level 1 (670 m).

##### Comments.

Collected on substrates of gravel, macrophytes, and leaf packs (June 2018, rainy season).

#### ﻿Genus Helichus

Taxon classificationAnimaliaColeopteraDryopidae

Erichson, 1847

F0D01C05-C954-5701-ACDF-84E0E108748F

##### Note.

This genus is found throughout the Oriental, Nearctic, and Neotropical regions, with 32 species described (Kodada and Jäch 2005).

#### ﻿ Helichus suturalis

Taxon classificationAnimaliaColeopteraDryopidae

LeConte, 1852

3A42B449-9C2E-5B67-ADAB-0E71B3260855

##### Distribution.

United States, Mexico (Chiapas, Durango, Hidalgo), Guatemala, Paraguay (Brown 1972a; Arce-Pérez et al. 2010; Shepard and Aguilar-Julio 2010). The known altitude records of the species are 1,590 and 2,438 m (Brown 1972a; Arce-Pérez et al. 2010). Herein, specimens were found at levels 1 (670 m), 2 (934 m), and 3 (1126–1194 m).

##### Comments.

Collected on substrates of gravel, macrophytes, and leaf packs, throughout the sampling period (February 2018 through February 2019, dry and rainy season).

### ﻿Family Dytiscidae Leach, 1815

#### ﻿Genus Bidessonotus

Taxon classificationAnimaliaColeopteraDytiscidae

Régimbart, 1895

4EA85577-6394-5BC0-87BC-01A6DF2EBD42

##### Note.

This is one of the largest dytiscid genera in the New World, comprising 36 species (Nilsson and Hájek 2020), with seven species recorded from Mexico (Arce-Pérez and Roughley 1999; Nilsson and Hájek 2020).

#### ﻿ Bidessonotus championi

Taxon classificationAnimaliaColeopteraDytiscidae

J. Balfour-Browne, 1947

2FA1A6EA-7B84-54D9-B556-9F2B6FDF1D7C

##### Distribution.

Mexico (new country record, Chiapas), Guatemala, Honduras, Nicaragua, Costa Rica (Balfour-Browne 1947; Miller 2016; Nilsson and Hájek 2020). The species has been recorded from an altitude of ca. 122 m (Balfour-Browne 1947), herein we recorded the species at level 4 (1,619 m).

##### Comments.

Collected on macrophytes (February 2018, dry season).

#### ﻿Genus Clarkhydrus

Taxon classificationAnimaliaColeopteraDytiscidae

Fery & Ribera, 2018

4D49998F-429E-5D2F-9905-2F816244F519

##### Note.

This genus has a Nearctic and Neotropical distribution and comprises 10 species, seven of which have been recorded in Mexico (Nilsson and Hájek 2020).

#### ﻿ Clarkhydrus

Taxon classificationAnimaliaColeopteraDytiscidae

sp.

3D63C3D6-5CA1-5462-B0FC-B972F5491303

##### Comments.

This species was collected at levels 4 (rivers 1 and 2, 1,448–1,619 m) and 5 (river 1, 1,763 m) on substrates of macrophytes and leaf packs, and was present throughout sampling months (February 2018 through February 2019, dry and rainy season). Specimens did not match known described species of the genus; however, they are close to *C.decemsignatus*, yet male genital morphology differs.

#### ﻿Genus Copelatus

Taxon classificationAnimaliaColeopteraDytiscidae

Erichson, 1832

F872716A-DAD3-5FBB-AE0C-E482017BBCFF

##### Note.

This genus has a cosmopolitan distribution and comprises 454 species (Nilsson and Hájek 2020), 14 of which have been recorded in Mexico (Arce-Pérez and Roughley 1999; Nilsson and Hájek 2020).

#### ﻿ Copelatus distinctus

Taxon classificationAnimaliaColeopteraDytiscidae

Aubé, 1838

97B12837-0E6D-5BB4-8921-34246C321E15

##### Distribution.

United States, Mexico (Baja California, Chiapas, Guanajuato, Jalisco, Morelos, Oaxaca, Puebla, Sonora), Guatemala (Young 1963; Arce-Pérez and Roughley 1999; Zaragoza-Caballero et al. 2019; Nilsson and Hájek 2020). This species has been recorded from moderate elevations (Young 1963) and 1,706 m (Miller and Bergsten 2014), herein it was found at levels 3 (1,126–1,194 m), 4 (1,448–1,619 m), and 5 (1,126–1,776 m).

##### Comments.

Collected on substrates of macrophytes and leaf packs, through all months of sampling (February 2018 through February 2019, dry and rainy season); also collected with a bucket light trap.

#### ﻿Genus Ilybiosoma

Taxon classificationAnimaliaColeopteraDytiscidae

Crotch, 1873

447C623B-6804-5417-BC17-60AEEE64FEB7

##### Note.

This is a cosmopolitan genus that includes 17 species (Nilsson and Hákej 2020), five of them recorded in Mexico (Arce-Pérez and Roughley 1999; Nilsson and Hájek 2020).

#### ﻿ Ilybiosoma flohrianum

Taxon classificationAnimaliaColeopteraDytiscidae

(Sharp, 1887)

4CBCA516-981E-5350-88C8-60C9094603CD

##### Distribution.

Mexico (Estado de México; Chiapas, new state record; Morelos) (Zaragoza-Caballero et al. 2019; Nilsson and Hájek 2020). There are no published records of altitude for the species; herein, the species was found at levels 4 (1,619 m) and 5 (1,776 m).

##### Comments.

Specimens were found on leaf packs (May 2018, rainy season).

#### ﻿Genus Laccophilus

Taxon classificationAnimaliaColeopteraDytiscidae

Leach, 1815

B8A0242D-DF31-58EA-9C84-81E206F40919

##### Note.

This cosmopolitan genus is the largest of the subfamily Laccophilinae, with 285 species (Nilsson and Hákej 2020), 26 of which are recorded from Mexico (Arce-Pérez and Roughley 1999; Nilsson and Hájek 2020).

#### ﻿ Laccophilus proximus

Taxon classificationAnimaliaColeopteraDytiscidae

Say, 1823

2576A326-A1FB-5F85-932B-B525E050E318

##### Distribution.

United States, Mexico (Campeche, Chiapas, Coahuila, Oaxaca, San Luis Potosí, Tabasco, Tamaulipas, Yucatán, Veracruz), Belize, Guatemala, Costa Rica, Bahamas, Cuba, Puerto Rico, Guadeloupe (Scheer and Thomaes 2018; Nilsson and Hájek 2020). It has been recorded at altitudes between 14 and 2,438 m (Scheer and Thomaes 2018). In this study, it was collected between 1,126 and 1,723 m.

##### Comments.

Specimens were found on leaf packs (February and March 2018).

#### ﻿Genus Liodessus

Taxon classificationAnimaliaColeopteraDytiscidae

Guignot, 1939

13D6300F-B333-5A09-8332-935F0FDF3C5C

##### Note.

This genus is distributed in North and South America, Africa, and Fiji (Miller and Bergsten 2016) and comprises 40 species (Nilsson and Hájek 2020), with four species recorded from Mexico (Arce-Pérez and Roughley 1999; Nilsson and Hájek 2020).

#### ﻿ Liodessus affinis

Taxon classificationAnimaliaColeopteraDytiscidae

(Say, 1823)

F4C050E8-1785-5519-9470-CFB55B891E9D

##### Distribution.

Canada, United States, Mexico (Baja California; Estado de México; Chiapas, new state record) (Arce-Pérez and Roughley 1999; Nilsson and Hájek 2020). No specific data about altitudinal distribution were found, herein this species was collected at 1,448 m.

##### Comments.

Collected on macrophytes and leaf packs (February and March 2018, dry season).

#### ﻿Genus Neoclypeodytes

Taxon classificationAnimaliaColeopteraDytiscidae

Young, 1967

AC179384-82E1-519D-BDFF-675B4C0A1226

##### Note.

This genus is present from southwestern Canada south through western United States and Mexico, with a few species in Panama and one in Jamaica (Miller and Bergsten 2016; Nilsson and Hájek 2020). It comprises 27 species (Nilsson and Hájek 2020), 15 of which are present in Mexico (Arce-Pérez and Roughley 1999; Arce-Pérez and Novelo-Gutiérrez 2015; Nilsson and Hájek 2020).

#### ﻿ Neoclypeodytes fryii

Taxon classificationAnimaliaColeopteraDytiscidae

(Clark, 1862)

3E0AD53F-95E6-573A-8867-223FB0F58C15

##### Distribution.

United States, Mexico (Baja California; Chiapas, new state record; Guanajuato; Oaxaca), Guatemala (Miller 2001; Nilsson and Hájek 2020). This species was previously recorded at an altitudinal range between 853 and 1,524 m (Miller 2001). In this study, the species was collected at level 4 (rivers 1 and 2, 1,425–1,619 m).

##### Comments.

Collected on macrophytes and leaf packs, throughout the sampling period (February 2018 through February 2019, dry and rainy season).

#### ﻿Genus Platambus

Taxon classificationAnimaliaColeopteraDytiscidae

Thomson, 1859

5CA433CA-8C55-5177-8819-9FB399E1377D

##### Note.

This genus is distributed in the Nearctic, Neotropical, Palearctic, and Oriental regions, with 67 species (Miller and Bergsten 2016; Nilsson and Hájek 2020), six of which are recorded from Mexico (Arce-Pérez and Roughley 1999; Nilsson and Hájek 2020).

#### ﻿ Platambus americanus

Taxon classificationAnimaliaColeopteraDytiscidae

(Aubé, 1838)

3CAD434A-1F58-5EF2-87C3-10206BF449D1

##### Distribution.

Mexico (Chiapas, new state record; Oaxaca), Guatemala, El Salvador (Arce-Pérez and Roughley 1999; Larson et al. 2000; Hendrich et al. 2018; Nilsson and Hájek 2020). Previous altitudinal records are between 1,950 and 2,743 m (Hendrich et al. 2018), while in the present study the species ranged from levels 4 (river 2, 1,619m) to 4 (river 2, 1,776 m).

##### Comments.

Collected on macrophytes and leaf packs, throughout the sampling period (February 2018 through February 2019, dry and rainy season); also collected with a bucket light trap.

#### ﻿Genus Rhantus

Taxon classificationAnimaliaColeopteraDytiscidae

Dejean, 1833

059BB2BA-3757-569F-BAD2-A0B45894D3B1

##### Note.

This is a cosmopolitan genus with 90 species (Nilsson and Hájek 2020), four of which are recorded in Mexico (Arce-Pérez and Roughley 1999; Nilsson and Hájek 2020).

#### ﻿ Rhantus gutticollis

Taxon classificationAnimaliaColeopteraDytiscidae

(Say, 1830)

DE4ED9D3-10C0-5718-8E67-CB756D152313

##### Distribution.

Canada, United States, Mexico (Baja California, Coahuila, Colima, Chiapas, Chihuahua, Ciudad de México, Durango, Estado de México, Guanajuato, Hidalgo, Jalisco, Michoacán, Morelos, Nayarit, Nuevo Léon, Oaxaca, Puebla, Querétaro, San Luis Potosí, Sinaloa, Sonora, Tamaulipas, Veracruz, Zacatecas), Guatemala, Honduras, Nicaragua, Costa Rica (Blackwelder 1944; Zimmerman and Smith 1975; Balke 1992; Arce-Pérez and Roughley 1999; Larson et al. 2000; Zaragoza-Caballero et al. 2019; Nilsson and Hájek 2020). This species has been previously recorded from 0 to 2,250 m (Blanco-Aller and Régil 2016), herein it was collected at level 5 (river 2, 1,776 m).

##### Comments.

Collected on leaf packs (May 2018, rainy season).

#### ﻿Genus Thermonectus

Taxon classificationAnimaliaColeopteraDytiscidae

Dejean, 1833

BEB4121E-D4EA-5449-8E60-36E9E13ACE2C

##### Note.

This genus is distributed across the Americas and comprises 20 species and two subspecies (Nilsson and Hajék 2020), with eight species recorded from Mexico (Arce-Pérez and Roughley 1999; Nilsson and Hájek 2020).

#### ﻿ Thermonectus nigrofasciatus

Taxon classificationAnimaliaColeopteraDytiscidae

(Aubé, 1838)

92154E35-7D57-503B-8164-ED32A254F61E

##### Distribution.

Mexico (Ciudad de México, Chiapas, Durango, Estado de México, Guanajuato, Hidalgo, Morelos, Oaxaca, Puebla, San Luis Potosí) (Arce-Pérez and Roughley 1999; Zaragoza-Caballero et al. 2019; Nilsson and Hájek 2020). This species was collected at level 5 (river 2, 1,776 m).

##### Comments.

Collected on leaf packs (May 2018, rainy season).

#### ﻿Genus Uvarus

Taxon classificationAnimaliaColeopteraDytiscidae

Guignot, 1939

FE695D25-88AE-501B-B94C-1104F7BC2E13

##### Note.

This genus is distributed worldwide and contains 65 species, nine of which are present in Mexico (Larson et al. 2000; Miller and Bergsten 2016; Nilsson and Hájek 2020).

#### ﻿ Uvarus subornatus

Taxon classificationAnimaliaColeopteraDytiscidae

(Sharp, 1882)

5EF50FBB-7BF3-5895-A829-7715880587BE

##### Distribution.

Mexico (Chiapas, Oaxaca), Guatemala (Arce-Pérez and Roughley 1999; Nilsson and Hájek 2020). No previous altitudinal records for this species were found. In the present study, this species was found only at level 5 (river 2, 1,776 m).

##### Comments.

Collected on leaf packs (May 2018, rainy season).

### ﻿Family Elmidae Curtis, 1830

#### ﻿Genus Austrolimnius

Taxon classificationAnimaliaColeopteraElmidae

Carter & Zeck, 1929

E36F95A3-BEC2-59D5-8118-DE1BC064D835

##### Note.

This genus occurs in the Australasian and Neotropical regions, with more than 100 described species (Manzo 2005, 2007; Jäch et al. 2016). Twenty species of this genus have been recorded in the Americas, from northern Mexico through southeastern Argentina (Hinton 1971; Manzo 2007), with four species recorded from Mexico (Santiago-Fragoso and Spangler 1995; Jäch et al. 2016).

#### ﻿ Austrolimnius formosus

Taxon classificationAnimaliaColeopteraElmidae

(Sharp, 1882)

5E190E54-E91B-5133-967D-ED71FE519709

##### Distribution.

Mexico (Chiapas, Morelos, Guerrero), Belize, Guatemala, Nicaragua, Costa Rica, Panama, Colombia, Venezuela, Peru, Brazil, Argentina (Sharp 1882; Hinton 1940b, 1941, 1971; Blackwelder 1944; Shepard 2004; Manzo 2007; Passos et al. 2009; Manzo and Archangelsky 2012; Miranda et al. 2012; González-Córdoba et al. 2016, 2020). Previous altitudinal records of *A.formosus* are from 600 m and 2,438 m (Hinton 1940b). In this study, the species was present in all sampled levels (670–1,776 m).

##### Comments.

Collected on substrates of gravel, macrophytes, and leaf packs, throughout the sampling period (February 2018 through February 2019, dry and rainy season).

#### ﻿ Austrolimnius sulcicollis

Taxon classificationAnimaliaColeopteraElmidae

(Sharp, 1882)

94FE0278-E993-5CBC-9D37-1D1119BC2748

##### Distribution.

Mexico (Chiapas, Guerrero), Guatemala, Costa Rica, Panama, Colombia, Venezuela, French Guiana, Ecuador, Peru (Sharp 1882; Hinton 1940b, 1941, 1971; Blackwelder 1944; González-Cordoba et al. 2020). *Austrolimniussulcicollis* has been previously recorded from altitudes of 600 m and 2,438 m (Hinton 1940b). Herein, this species was collected in all sampling levels (670–1,776 m).

##### Comments.

Collected on substrates of gravel, macrophytes, and leaf packs, throughout the sampling period (February 2018 through February 2019, dry and rainy season).

#### ﻿Genus Cylloepus

Taxon classificationAnimaliaColeopteraElmidae

Erichson, 1847

6CC6C5F2-E07C-53A8-A688-C4C4A6466676

##### Note.

This is the elmid genus with most species in the American continent, with 52 species and 2 subspecies currently known to this region (Segura et al. 2013; Jäch et al. 2016; Silva-Polizei and Barclay 2019), and eight species recorded in Mexico (Santiago-Fragoso and Spangler 1995; Jäch et al. 2016).

#### ﻿ Cylloepus atys

Taxon classificationAnimaliaColeopteraElmidae

Hinton, 1946

E7DD8A20-B491-5833-B145-0AAF0E67C213

##### Distribution.

Mexico (new country record, Chiapas), Peru (Hinton 1946). Previous altitudinal records are from approximately 500 m (Hinton 1940a). In this study, the species was collected at levels 1 (670 m), 2 (934 m), 3 (river 1, 1,126 m), 4 (river 2, 1,619 m), and 5 (rivers 1 and 2, 1,763–1,776 m).

##### Comments.

Collected on substrates of gravel, macrophytes, and leaf packs, through most of the sampling months (except March, July, and September 2018, dry and rainy season).

#### ﻿Genus Heterelmis

Taxon classificationAnimaliaColeopteraElmidae

Sharp, 1882

CB52F79B-DE21-596A-AFC2-0D9C9B1EA982

##### Note.

This is a New World genus that comprises 22 species (Silva-Polizei 2018), seven of which are present in Mexico (Santiago-Fragoso and Spangler 1995; Jäch et al. 2016).

#### ﻿ Heterelmis glabra

Taxon classificationAnimaliaColeopteraElmidae

(Horn, 1870)

C568188E-68FD-566D-AB89-209C66E3F592

##### Distribution.

Mexico (Chiapas, Estado de México, Hidalgo, Jalisco, Morelos, Nayarit, Oaxaca, Tamaulipas, Veracruz), Belize, Nicaragua, Costa Rica (Santiago-Fragoso and Spangler 1995; Jäch et al. 2016). This species was previously recorded from 1,066 m and 1,219 m (Hinton 1940b). Herein, this species was found at all sampled levels (670–1,776 m).

##### Comments.

Collected on substrates of gravel, macrophytes, and leaf packs, through all sampling months (February 2018 through February 2019, dry and rainy season).

#### ﻿ Heterelmis obesa

Taxon classificationAnimaliaColeopteraElmidae

Sharp, 1882

18A1EE08-C805-5F40-AC98-50D69570101E

##### Distribution.

Mexico (Chiapas, Durango, Estado de México, Hidalgo, Morelos, Oaxaca, Veracruz), Guatemala, Costa Rica, Nicaragua, Peru (Sharp 1882; Hinton 1940b; Blackwelder 1944; Spangler 1966; Brown 1972b; Jäch et al. 2016). This species was previously recorded from 1,463 m and 2,438 m (Hinton 1940b). In this study, the species was found at all sampled levels (670–1,776 m).

##### Comments.

Collected on substrates of gravel, macrophytes, and litter, throughout all sampling months (February 2018 through February 2019, dry and rainy season).

#### ﻿ Heterelmis obscura

Taxon classificationAnimaliaColeopteraElmidae

Sharp, 1882

8648C967-9E2B-54EB-BF4A-BAC81D9A8838

##### Distribution.

Mexico (Chiapas, Colima, Estado de México, Morelos, Nuevo León, Oaxaca, San Luis Potosí, Veracruz), Guatemala, Costa Rica, Colombia, Peru, Brazil (Sharp 1882; Grouvelle 1889; Hinton 1940b; Blackwelder 1944; Brown 1972b; Santiago-Fragoso and Spangler 1995; Passos et al. 2009; Segura et al. 2013; Jäch et al. 2016). Previous altitudinal records of *H.obscura* are from 1,463 m and 2,438 m (Hinton 1940b). In this study, the species was found in all sampled levels (670–1,776 m).

##### Comments.

Collected on substrates of gravel, macrophytes, and leaf packs, throughout sampling months (February 2018 through February 2019, dry and rainy season).

#### ﻿ Heterelmis simplex

Taxon classificationAnimaliaColeopteraElmidae

Sharp, 1882

43B341A8-ED39-50CE-9F78-8D1FA705A2CD

##### Distribution.

Mexico (Chiapas, Morelos), Guatemala, Costa Rica, Peru, Trinidad and Tobago (Santiago-Fragoso and Spangler 1995; Segura et al. 2013; Jäch et al. 2016). No previous altitudinal records were found. This species was collected at all sampled levels (670–1,776 m).

##### Comments.

Collected on substrates of gravel, macrophytes, and leaf packs, throughout the sampling months (February 2018 through February 2019, dry and rainy season).

#### ﻿Genus Hexacylloepus

Taxon classificationAnimaliaColeopteraElmidae

Hinton, 1940b

7454EC8C-9C82-5F72-9445-6D7DDE6C42F6

##### Note.

This genus is distributed in the southwestern United States and the Neotropical region, with 25 described species (Jäch et al. 2016; Silva-Polizei et al. 2020), seven of which are recorded from Mexico (Santiago-Fragoso and Spangler 1995; Jäch et al. 2016; Silva-Polizei et al. 2020).

#### ﻿ Hexacylloepus metapa

Taxon classificationAnimaliaColeopteraElmidae

Silva-Polizei, Barclay & Bispo, 2020

9FCD1985-0E35-5C72-90B1-E5FCF1CAD61C

##### Distribution.

Mexico (new country record, Chiapas), Guatemala (Silva-Polizei et al. 2020). There are no previous records of altitude for *H.metapa*, herein the species was collected at levels 1 (670 m), 2 (934 m), 3 (1,126–1,194 m), 4 (river 2, 1,619 m), and 5 (1,763–1,776 m).

##### Comments.

Collected on substrates of gravel, macrophytes, and leaf packs, throughout sampling months (February 2018 to February 2019, dry and rainy season).

#### ﻿Genus Hexanchorus

Taxon classificationAnimaliaColeopteraElmidae

Sharp, 1882

D928F404-AB1F-532E-BA40-D419511D2F6B

##### Note.

This is a New World genus and comprises 21 species, with three recorded from Mexico (Santiago-Fragoso and Spangler 1995; Jäch et al. 2016).

#### ﻿ Hexanchorus usitatus

Taxon classificationAnimaliaColeopteraElmidae

Spangler & Santiago-Fragoso, 1992

3596A5AC-ABA3-5D48-BD19-0B128CC15FFC

##### Distribution.

Mexico (new country record, Chiapas), Nicaragua, Costa Rica, Panama.

The known altitudinal record of *H.usitatus* was 1,075 m (Spangler and Santiago-Fragoso 1992). Herein, the species was found from levels 1 (670 m) through 3 (1,126–1,194 m).

##### Comments.

Collected on substrates of gravel, macrophytes, and leaf packs, throughout sampling months (February 2018 through February 2019, dry and rainy season); also collected with a bucket light trap.

#### ﻿Genus Huleechius

Taxon classificationAnimaliaColeopteraElmidae

Brown, 1981

C305923D-BAF2-536A-9CB2-7BEF236CC943

##### Note.

This is a North American genus and includes three species (Jäch et al. 2016), with two recorded from Mexico (Santiago-Fragoso and Spangler 1995; Jäch et al. 2016).

#### ﻿ Huleechius spinipes

Taxon classificationAnimaliaColeopteraElmidae

(Hinton, 1934)

28B19FBE-CE94-5527-9BE5-18C3C737921C

##### Distribution.

Mexico (Baja California; Chiapas, new state record; Coahuila; Estado de México; Guerrero; Jalisco; Nuevo León; Oaxaca; Tabasco; Veracruz) (Santiago-Fragoso and Spangler 1995; Jäch et al. 2016). A previous altitudinal record of *H.spinipes* is from 1,524 m (Hinton 1940b). In this study, the species was found at levels 1 (670 m), 2 (934 m), 3 (1,126–1,194 m), 4 (river 2, 1,619 m), and 5 (1,763–1,776 m).

##### Comments.

Collected on substrates of gravel, macrophytes, and leaf packs, throughout sampling months (February 2018 through February 2019, dry and rainy season).

#### ﻿Genus Macrelmis

Taxon classificationAnimaliaColeopteraElmidae

Motschulsky, 1859

735FF76D-341F-582F-998C-3F33DA0AFC68

##### Note.

This is a Nearctic and Neotropical genus, distributed from southern United States to South America, and comprises 49 species, 10 of which have been recorded from Mexico (Hinton 1940b; Passos et al. 2015; Jäch et al. 2016).

#### ﻿ Macrelmis graniger

Taxon classificationAnimaliaColeopteraElmidae

(Sharp, 1882)

AF32F797-55F1-5867-AB39-36D6E0761D09

##### Distribution.

Mexico (Chiapas, Estado de México, Morelos, Oaxaca), Guatemala, Costa Rica, Nicaragua, Peru (Santiago-Fragoso and Spangler 1995; Segura et al. 2013; Jäch et al. 2016). Previous altitudinal records of *M. graniger* are from 1,219 and 1,706 m (Hinton 1940b). Herein, this species was collected at level 1 (670 m), 2 (934 m), 3 (1,126–1,194 m), 4 (river 2, 1,619 m), and 5 (1,763–1,776 m).

##### Comments.

Collected on substrates of gravel, macrophytes, and leaf packs, throughout sampling months (February 2018 through February 2019, dry and rainy season).

#### ﻿ Macrelmis leonilae

Taxon classificationAnimaliaColeopteraElmidae

Spangler & Santiago-Fragoso, 1986

F23E5A6B-24A7-537E-B6F5-E07CF5AFC451

##### Distribution.

Mexico (Chiapas, Guerrero, Morelos, Oaxaca, Veracruz), Guatemala, Honduras, Nicaragua, Costa Rica, Peru (Santiago-Fragoso and Spangler 1995; Segura et al. 2013). A previous altitudinal record of *M. leonilae* is 1,075 m (Spangler and Santiago-Fragoso 1986). Herein, this species was collected at level 1 (670 m), 2 (934 m), 3 (1,126–1,194 m), 4 (river 2, 1,619 m), and 5 (1,763–1,776 m).

##### Comments.

Collected on substrates of gravel, macrophytes, and leaf packs, throughout sampling months (February 2018 through February 2019, dry and rainy season).

#### ﻿ Macrelmis

Taxon classificationAnimaliaColeopteraElmidae

sp.

7FD32902-3DF2-5C24-822E-886B12FCBC24

##### Comments.

This species was collected at level 5 (river 2, 1,776 m) on substrates of macrophytes and leaf packs, and was present throughout sampling months (February 2018 through February 2019, dry and rainy season). Specimens, including males, did not match known described species of the genus, although they are similar to *M. leonilae*. Male parameres of the specimens, in dorsal view, are slightly wider from the base to the apical portion, while in *M. leonilae* they are wider through the basal half.

#### ﻿Genus Microcylloepus

Taxon classificationAnimaliaColeopteraElmidae

Hinton, 1935

B81E4206-99F2-5D68-B1F9-79A098502767

##### Note.

*Microcylloepus* is widely distributed in the New World and comprises 30 species (Silva-Polizei 2018), five of them recorded from Mexico (Santiago-Fragoso and Spangler 1995; Jäch et al. 2016).

#### ﻿ Microcylloepus inaequalis

Taxon classificationAnimaliaColeopteraElmidae

(Sharp, 1882)

E4D5E881-60F4-5724-86F4-12AC07A940EB

##### Distribution.

Mexico (Chiapas, Estado de Mexico, Morelos, Veracruz), Guatemala, Nicaragua, Costa Rica, Panama, Paraguay, Brazil (Santiago-Fragoso and Spangler 1995; Segura et al. 2013; Jäch et al. 2016). Previous altitudinal records of *M. inaequalis* are at 1,463 m and 1,525 m (Hinton 1940c). Herein, this species was found at all sampled levels (670–1,776 m).

##### Comments.

Collected on substrates of gravel, macrophytes, and leaf packs, throughout sampling months (February 2018 through February 2019, dry and rainy season).

#### ﻿ Microcylloepus troilus

Taxon classificationAnimaliaColeopteraElmidae

Hinton, 1940

3775E0BB-1057-5073-A487-73BC80533EA0

##### Distribution.

Mexico (Chiapas, Estado de Mexico). Previous altitudinal records of *M. troilus* are from 1,707 to 2,286 m (Hinton 1940b). In this study, *M. troilus* was found at all sampled levels (670–1,776 m).

##### Comments.

Collected on substrates of gravel, macrophytes, and leaf packs, throughout sampling months (February 2018 through February 2019, dry and rainy season).

#### ﻿ Microcylloepus

Taxon classificationAnimaliaColeopteraElmidae

sp.

772D47EE-0E39-5F02-B838-73DD86BB3026

##### Comments.

This species was collected at levels 1 (670 m), 2 (934 m), 3 (1,126–1,194 m), 4 (river 2, 1,619 m), and 5 (river 2, 1,776 m) on substrates of gravel, macrophytes, and leaf packs, throughout sampling months (February 2018 through February 2019, dry and rainy season). Specimens, including males, did not match exactly known described species of the genus, being close to *M. angustus*. Male genitalia of the specimens have the medium lobe slightly wider than *M. angustus*.

#### ﻿Genus Neoelmis

Taxon classificationAnimaliaColeopteraElmidae

Musgrave, 1935

ACAA5F63-B3D0-526B-A917-9539B842EDA6

##### Note.

This genus is distributed across the American continent and has 50 described species (Jäch et al. 2016), five of them recorded from Mexico (Santiago-Fragoso and Spangler 1995; Jäch et al. 2016).

#### ﻿ Neoelmis apicallis

Taxon classificationAnimaliaColeopteraElmidae

(Sharp, 1882)

7375B97D-7556-57CE-BBAD-E547CD72F9E1

##### Distribution.

Mexico (Chiapas, Estado de México, Morelos, San Luis Potosí, Tamaulipas), Guatemala, Costa Rica (Santiago-Fragoso and Spangler 1995; Segura et al. 2013; Jäch et al. 2016). Previous altitudinal records were at 137 m and 1,463 m (Hinton 1940b). In this study, the species was found at levels 1 (670 m), 2 (934 m), and 3 (1,126–1,194 m).

##### Comments.

Collected on substrates of gravel, macrophytes, and leaf packs, in about half of the sampling period (February to May, and August 2018, and February 2019, dry and rainy season).

#### ﻿Genus Onychelmis

Taxon classificationAnimaliaColeopteraElmidae

Hinton, 1941

9B150D1F-1057-5F14-AE83-2BE338CB0F42

##### Note.

This genus is distributed in Central and South America, contains eight described species (Linský et al. 2021), and this study provides the northernmost point of its range.

#### ﻿ Onychelmis longicollis

Taxon classificationAnimaliaColeopteraElmidae

(Sharp, 1882)

885FF937-A3DD-5A8B-B63D-A99723A70969

##### Distribution.

Mexico (new country record, Chiapas), Nicaragua, Panama, Colombia (González-Córdoba et al. 2016; Linský et al. 2021). Previous altitudinal records were from 1,219 to 1,828 m (González-Córdoba et al. 2016; Linský et al. 2021). In the present study, the species was found at levels 1 (670 m), 2 (934 m), and 3 (1,126–1,194 m).

##### Comments.

Collected on substrates of gravel, macrophytes, and leaf packs, during three months of the sampling period (February, April, and May 2018, dry and rainy season).

#### ﻿Genus Phanocerus

Taxon classificationAnimaliaColeopteraElmidae

Sharp, 1882

A24D09F6-3B13-5D61-9C92-C48739D7EF45

##### Note.

This genus is distributed from North America through northern South America, with six described species (Jäch et al. 2016), one recorded in Mexico (Santiago-Fragoso and Spangler 1995; Jäch et al. 2016).

#### ﻿ Phanocerus clavicornis

Taxon classificationAnimaliaColeopteraElmidae

Sharp, 1882

FC07EF87-5246-56C4-BB05-FD2DD3F7CCDA

##### Distribution.

United States, Mexico (Chiapas, Colima, Guerrero, Hidalgo, Nuevo León, Puebla, Querétaro, San Luis Potosí, Tamaulipas, Veracruz), Belize, Guatemala, Honduras, Costa Rica, Panama, Venezuela, Brazil, Cuba, Jamaica, Haiti, Dominican Republic, Puerto Rico (Spangler and Santiago-Fragoso 1992; Segura et al. 2013; Jäch et al. 2016). This species was previously recorded from an altitudinal range of 88–549 m (Hinton 1940b; Spangler and Santiago-Fragoso 1992). Herein, the species was found at levels 1 (670 m), 3 (1,126–1,194), and 4 (1,448–1,619 m).

##### Comments.

Collected on substrates of gravel, macrophytes, and leaf packs through four months of the sampling period (May, June, July, and August 2018, rainy season); also collected with a bucket light trap.

#### ﻿Genus Xenelmis

Taxon classificationAnimaliaColeopteraElmidae

Hinton, 1936

9E63C392-AAF4-5394-BF26-26DC81A14195

##### Note.

This is a New World, mostly Neotropical genus with 11 described species (Jäch et al. 2016), two of them recorded from Mexico (Santiago-Fragoso and Spangler 1995; Sampaio et al. 2015; Jäch et al. 2016).

#### ﻿ Xenelmis bufo

Taxon classificationAnimaliaColeopteraElmidae

(Sharp, 1882)

9F8ACADD-7E1A-5568-A591-5F6155AA5555

##### Distribution.

Mexico (Chiapas, Colima, Guerrero, Morelos), Belize, Panama, Venezuela (Segura et al. 2013; Jäch et al. 2016). A previous altitudinal record of *X.bufo* is from 1,219 m (Hinton 1940b). In this study, the species was found at all sampling levels (670–1,776 m).

##### Comments.

Collected on substrates of gravel, macrophytes, and leaf packs, throughout the sampling months (February 2018 through February 2019, dry and rainy season).

### ﻿Family Epimetopidae Zaitzev, 1908

#### ﻿Genus Epimetopus

Taxon classificationAnimaliaColeopteraEpimetopidae

Lacordaire, 1854

0AE7AE4E-1F09-5039-9ED4-30A79A8126A7

##### Note.

This genus is distributed across the Nearctic and Neotropical region, with 56 species described (Perkins 2012), eight of them recorded from Mexico (Arce-Pérez and Morón 2011; Perkins 2012).

#### ﻿ Epimetopus thermarum

Taxon classificationAnimaliaColeopteraEpimetopidae

Schwarz & Barber, 1917

27F06666-803B-563F-B714-7BCB1E1AE8EC

##### Distribution.

United States, Mexico (Baja California Sur, Chiapas, Jalisco, Nayarit, Sinaloa, Sonora), Belize, Guatemala, Costa Rica, Panama, Venezuela (Perkins 2012).

This species was previously recorded at an altitudinal range of 5–914 m (Perkins 2012). In this study, the species was found in level 1 (670 m).

##### Comments.

Collected with a bucket light trap (June 2018, rainy season).

### ﻿Family Gyrinidae Latreille, 1810

#### ﻿Genus Gyretes

Taxon classificationAnimaliaColeopteraGyrinidae

Brullé, 1835

1EAC0A3D-EB21-544D-8E81-75B8BA36E33D

##### Note.

This genus comprises 79 species worldwide (Oygur and Wolfe 1991; Babin and Alarie 2014), seven of them recorded from Mexico (Arce-Pérez and Roughley 1999).

#### ﻿ Gyretes boucardi

Taxon classificationAnimaliaColeopteraGyrinidae

Sharp,1882

739254E8-6300-5733-AC47-E592245E64DA

##### Distribution.

Mexico (Chiapas, Durango, Tabasco, Veracruz), Costa Rica (Arce-Pérez and Roughley 1999; Blanco-Aller 2014). Previous altitudinal records of *G.boucardi* are from 0–125 m (Blanco-Aller 2014). In this study, the species was found at level 1 (670 m).

##### Comments.

Collected near substrate of macrophytes (October 2018, rainy season).

### ﻿Family Hydraenidae Mulsant, 1844

#### ﻿Genus Hydraena

Taxon classificationAnimaliaColeopteraHydraenidae

Kugelann, 1794

8E263306-54F3-5345-942E-087D3C5B8399

##### Note.

The genus occurs on all continents except Antarctica and comprises more than 990 species described (Trizzino et al. 2013), 36 of which are recorded from Mexico (Navarrete-Heredia and Quiroz-Rocha 2004).

#### ﻿ Hydraena

Taxon classificationAnimaliaColeopteraHydraenidae

sp.

E53C58D2-383D-5B10-A174-1A860B6B7678

##### Comments.

This species was collected at all sampled levels (670–1,776 m), on substrates of gravel, macrophytes, and leaf packs, throughout the sampling months (February 2018 through February 2019, dry and rainy season). Specimens were small and fragile, particularly males, and dissection was difficult, moreover genital morphology did not match species in keys, so genus-level identification was considered until further study; females were more abundant in collections.

### ﻿Family Hydrophilidae Latreille, 1802

#### ﻿Genus Tropisternus

Taxon classificationAnimaliaColeopteraHydrophilidae

Solier, 1834

3F87EED1-85BA-54F7-A191-22B857DD7ADE

##### Note.

This is a New World genus distributed from northern Canada to southern South America, comprising 60 described species (Hansen 1999; Short and Hebauer 2006; Spangler and Short 2008), 19 of them recorded from Mexico (Arce-Pérez and Morón 2011).

#### ﻿ Tropisternus fuscitarsis

Taxon classificationAnimaliaColeopteraHydrophilidae

Sharp, 1882

C4487880-A360-57B4-8737-124D3F8D2F36

##### Distribution.

Mexico (Chiapas, Colima, Distrito Federal, Jalisco, Estado de México, Michoacán, Morelos, Nayarit, Oaxaca, Puebla, Querétaro, San Luis Potosí, Sonora, Veracruz) (Arce-Pérez and Morón 2011). Previous altitudinal records of *T.fuscitarsis* are from 0–125 m (Blanco-Aller 2014). Herein, the species was found in level 2 (934 m).

##### Comments.

Collected with a bucket light trap (July and August 2018, rainy season).

### ﻿Family Lutrochidae Kasap & Crowson, 1975

#### ﻿Genus Lutrochus

Taxon classificationAnimaliaColeopteraLutrochidae

Erichson, 1847

F0AA60CA-9C80-5D7A-8E14-D211BF5EB901

##### Note.

This genus comprises 29 species and is distributed across the Nearctic and Neotropical region (Maier and Short 2013, 2014; Maier 2016), with three species recorded from Mexico (Arce-Pérez et al. 2010; Maier 2016).

#### ﻿ Lutrochus

Taxon classificationAnimaliaColeopteraLutrochidae

sp.

56F96AF5-C0F5-5BB8-8B47-DFD599554CA2

##### Comments.

This species was present at level 1 (670 m) and was collected on leaf packs (May 2018, rainy season). Specimens key out to an undescribed genus and species included in Maier (2016), an unpublished doctoral thesis, so a preliminary identification is maintained.

### ﻿Family Noteridae Thomson, 1860

#### ﻿Genus Notomicrus

Taxon classificationAnimaliaColeopteraLutrochidae

Sharp, 1882

2A722FBC-B615-5468-B8AB-793049D429AA

##### Note.

This genus comprises 15 species, 13 of them distributed in the New World (Baca et al. 2014; Guimarães and Ferreira 2019), and two of the latter species recorded from Mexico (Nilsson 2011).

#### ﻿ Notomicrus sharpi

Taxon classificationAnimaliaColeopteraLutrochidae

J. Balfour-Browne, 1939

D4708A3A-B23D-526C-B7D3-1F6ED5E9A3CB

##### Distribution.

United States, Mexico (Chiapas, new state record; Oaxaca; San Luis Potosí; Tamaulipas), Guatemala, Costa Rica, Panama, Bahamas, Cuba, Jamaica, Dominican Republic, Puerto Rico, Virgin Islands, Guadeloupe (Arce-Pérez and Roughley 1999; Nilsson 2011; Manuel 2015). This species was previously recorded from 0–500 m (Blanco-Aller 2015; Manuel 2015). Herein, the species was found at level 4 (river 2, 1,619 m).

##### Comments.

Collected on substrate of macrophytes (February 2018, dry season).

### ﻿Altitudinal distribution of the aquatic beetle fauna

**Figure 3.**
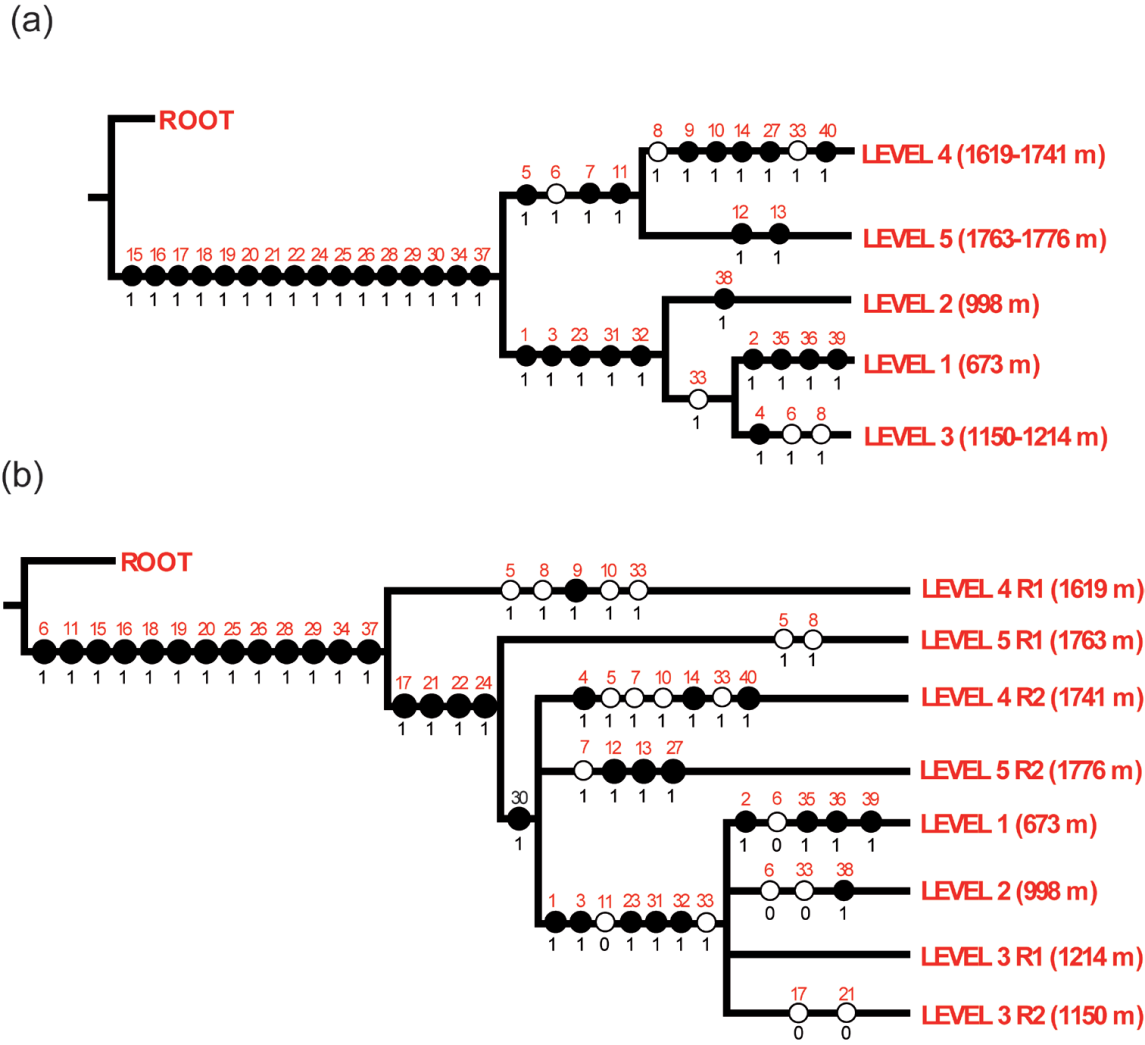
Parsimony Analysis of Endemicity (PAE) of the altitudinal levels and sites of the aquatic beetle fauna of Volcán Tacaná, Chiapas, Mexico **A** most parsimonious tree of the five sampling levels, with levels 3–5 considered each as a unit (number of steps = 43, consistency index (CI) = 93, retention index (RI) = 83) **B** strict consensus of the five most parsimonious trees of the five sampling levels, with levels 3–5 considered as two separate units each (number of steps = 53, consistency index (CI) = 75, retention index (RI) = 63). Red numbers = species (see Table 1); 1 = presence, 0 = absence, black circles = single event or first appearance, white circles = independent event or reversal (disappearance).

The aquatic beetle fauna from Volcán Tacaná is distributed throughout the sampled altitudinal gradient (670–1,776 m), however our initial hypothesis is that species distribution would not be homogeneous. We applied a Parsimony Analysis of Endemism (PAE) as a fast approach to detect a potential faunal partition, with a general finding of the three lower altitudinal levels grouping together (i.e., sharing similar species) and about 40% of the species with a widespread altitudinal distribution. A first PAE (Fig. 3A), including the five levels, each as a single unit, recovered a topology distinguishing two well-defined groups, one composed by the three lower levels (673, 998, and 1,150–1,214 m), and another composed by the two higher levels (1,619–1,741 m and 1,763–1,776 m). A second PAE (Fig. 3B), including each sampled river (i.e., rivers of levels 3–5 considered each as a unit) also recovered a group composed by the three lower levels (levels 1–3), nevertheless two rivers of levels 4 and 5 (i.e., R2 of levels 4 and 5, respectively) were recovered as closer to rivers from levels 1–3 than to other rivers of levels 4 and 5 (i.e., R1 of levels 4 and 5, respectively), yet support for the latter group (levels 1–3 + R2 of L4 and L5) is quite weak. This means that the next well supported group would be all rivers excluding river 1 of level 4.

**Figure 4.**
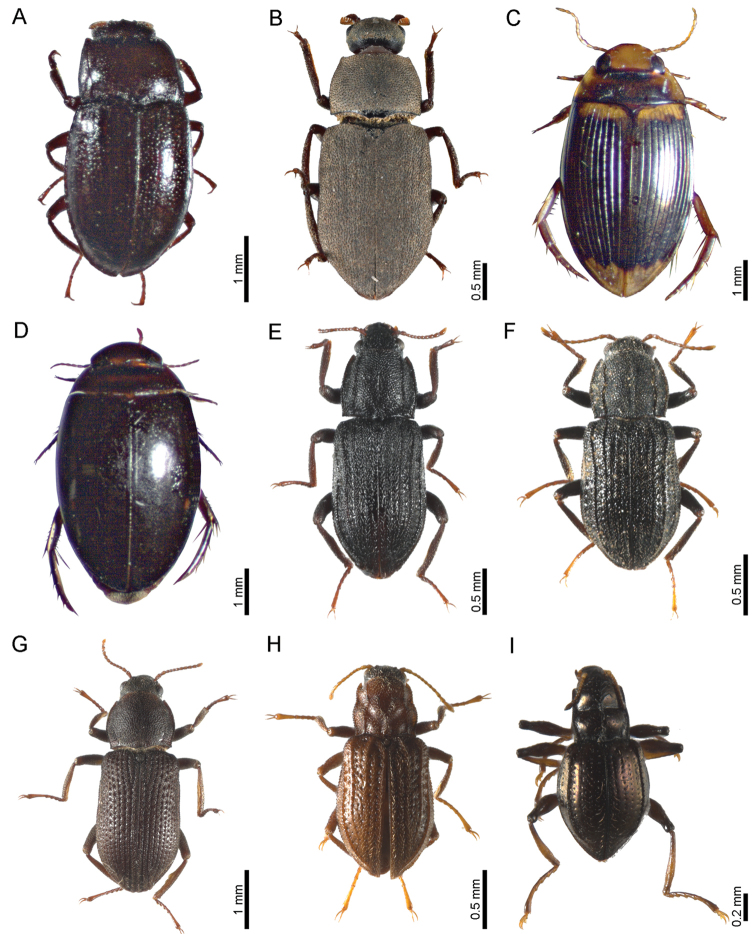
Habitus of representative species of the three most diverse aquatic beetle families from Volcán Tacaná, Chiapas, Mexico **A***Elmoparnuspandus* Spangler & Perkins, 1977 (Dryopidae) **B***Helichussuturalis* LeConte, 1852 (Dryopidae) **C***Copelatusdistinctus* Aubé, 1838 (Dytiscidae) **D***Platambusamericanus* (Aubé, 1838) (Dytiscidae) **E***Cylloepusatys* Hinton, 1946 (Elmidae) **F***Hexacylloepusmetapa* Silva-Polizei, Barclay & Bispo, 2020 (Elmidae) **G***Macrelmisleonilae* Spangler & Santiago-Fragoso, 1986 (Elmidae) **H***MicrocylloepusTroilus* Hinton, 1940 (Elmidae) **I***Onychelmislongicollis* (Sharp, 1882) (Elmidae).

The most diverse family was Elmidae (see some representatives on Fig. 4), with most species widespread along the five altitudinal levels, with the genera *Austrolimnius* (*A.formosus* and *A.sulcicollis*), *Xenelmis* (*X.bufo*), and *Heterelmis* (*H.glabra*, *H.obesa*, *H.obscura*, and *H.simplex*), occurring in all levels (except *H.simplex*, absent from R2 and R1 of levels 3 and 4, respectively). *Cylloepusatys* shares the same distribution pattern as *H.simplex*, while *Hexacylloepusmetapa* and *Huleechiusspinipes*, both occur in all altitudinal levels but are curiously absent from river 1 of level 4. *Macrelmisgraniger* and *M. leonilae* are present in all rivers, while *Macrelmis* sp. is present only in river 2 of level 5. *Microcylloepus* (*M. inaequalis*, *M. troilus*, and *M.* sp.) are present in all altitudinal levels, however *M. troilus* is absent in river 1 of level 4 and river 1 of level 5. *Phanocerusclavicornis* has a fragmented distribution, occurring in levels 1, 3, and 4, while *Hexanchorususitatus*, *Neoelmisapicalis*, and *Onychelmislongicollis* are present in all rivers from levels 1–3.

Dytiscidae (see some representatives on Fig. 4), the second most diverse family, is characteristic of the higher levels (i.e., levels 4 and 5), with all genera represented by only one species. *Copelatusdistinctus*, present in all rivers of levels 3–5, has the largest vertical distribution. *Platambusamericanus*, *Ilyobiosomaflohrianum*, *Laccophilusproximus*, and *Clarkhydrus* sp. are present in levels 3 and 4, however only *P.americanus* is present in all four rivers of these levels. *Bidessonotuschampioni*, *Liodessusaffinis*, *Uvarussubornatus*, and *Neoclypeodytesfryii* are present in level 4, nevertheless only the latter species occurs in both sampled rivers. *Rhantusgutticollis* and *Thermonectusnigrofasciatus* are only present in river 2 of the highest level.

Dryopidae (see some representatives in Fig. 4) is present in the three lowest levels, with *Dryopsmexicanus* and *Helichussuturalis* present in all rivers of such levels, while *Elmoparnuspandus* occurs only in level 1. *Epimetopusthermarum* (Epimetopidae), *Gyretesboucardi* (Gyrinidae), and *Lutrochus* sp. (Lutrochidae) occur only in level 1, while *Tropisternusfuscitarsis* (only recorded hydrophilid) is present in level 2, and *Notomicrussharpi* (only noterid) occurs only in river 2 of level 4. Hydraenidae (*Hydraena* sp.) was present in all sampled rivers.

### ﻿Biogeographical affinity of the aquatic beetle fauna

We attempt a general characterization of the fauna applying the criterion of Nearctic and Neotropical regions of Morrone (2006, 2017, 2019). Despite altitude of the sampled rivers, all species collected have distribution records within the Neotropical region (i.e., all levels present species of Neotropical affinity). About 60% of the species (24 out of 40) have a predominantly Neotropical distribution, while the remaining 40% (16 species) have a wide distribution in the New World, among which the elmids *Heterelmisglabra*, *H.obesa*, *H.obscura*, and *Huleechiusspinipes* occur in all altitudinal levels (670–1,776 m); *Helichussuturalis* (Dryopidae), *Neoelmisapicalis* (Elmidae), and *Tropisternusfuscitarsis* (Hydrophilidae), *Epimetopusthermarum* (Epimetopidae) occur only in the two lowest levels (670 and 998 m); while *Notomicrussharpi* (Noteridae) and the dytiscids *Laccophilusproximus*, *Liodessusaffinis*, *Neoclypeodytesfryii*, *Rhantusgutticollis*, and *Thermonectusnigrofasciatus* occurr only in the two highest levels (1,610–1,776 m). Other two species with Neotropical and Neartic records have a fragmented vertical distribution (the elmid *Phanocerusclavicornis*) or occur in the three highest levels (the dytiscid *Copelatusdistinctus*).

Among the 20 species of Elmidae, 14 occur only in the Neotropical region, while the remaining six, particularly those of *Heterelmis*, have a wide distribution (i.e., they occur in the Nearctic and Neotropical regions). Most dytiscid species, six out of 11, have a wide distribution through the Nearctic and Neotropics, while the other five occur only in the Neotropical region. Elmidae and Dysticidae have 80 and 50% of their distribution in the Brazilian subregions and the Mexican Transition Zone, respectively, with especial affinity to the Mesoamerican and Pacific domains. Dryopidae is represented by three species, two of them with records in the Neotropical region (Brazilian subregions and the Mexican Transition Zone) and one with Nearctic and Neotropical distribution. Gyrinidae (*Gyretesboucardi*), Hydraenidae (*Hydraena* sp.), and Lutrochidae (*Lutrochus* sp.), also have species with Neotropical affinity, whereas Epimotopidae (*Epimetopusthermarum*), Hydrophilidae (*Tropisternusfuscitarsis*), and Noteridae (*Notomicrussharpi*) have species with a wide distribution in the New World. The latter six families also have an affinity to the Brazilian subregions, particularly to the Pacific and Mesoamerican domains.

## ﻿Discussion

Aquatic beetles were present at the five sampling levels (L1, 673 m; L2, 998 m; L3, 1,150–1,214 m; L4, 1,619–1,741 m; and L5, 1,763–1,776m). This agrees with the widespread distribution of aquatic beetles, as well as their high capacity to inhabit different aquatic environments from sea level to mountains of 4,000 m high or more (Jäch and Balke 2008; White and Roughley 2008). Despite their broad presence in the volcano, aquatic beetle species were not distributed homogeneously along the altitudinal gradient, which is congruent with a high endemism in almost all families of this group, particularly those of lotic systems in warm climates (Jäch and Balke 2008).

Elmidae (riffle beetles) was the dominant group (20 spp.) and was present in all sampling levels. This coincides with previous findings in the Neotropics (e.g., Arias-Díaz et al. 2007; Huanachin-Quispe and Huamantico-Araujo 2018; Mosquera-Murillo and Sánchez-Vázquez 2018; Passos et al. 2018). General characteristics of the streams on a volcanic bedrock with a variety of substrates, such as gravel, leaf litter, logs, and aquatic macrophytes, probably contributed to maintain a high diversity of elmids as reported by Elliot (2008) and Mosquera-Murillo and Sánchez-Vázquez (2018). Species of the New World genera *Heterelmis*, *Macrelmis*, and *Microcylloepus*, and of the Neotropical *Austrolimnius* were present at all levels, while the Neotropical *Hexanchorus*, *Neoelmis*, and *Onychelmis* were restricted to levels 1–3.

Dytiscidae (predaceous diving beetles) was the second most diverse group (11 spp.) and was present mostly at levels 4 and 5, with only one species (*Copelatusdistinctus*) at levels 3–5. Three species, *Bidessonotuschampioni*, *Ilybiosomaflohrianum*, and *Uvarussubornatus* were only observed at L4 (R2, 1,619 m), while *Rhantusgutticollis* and *Thermonectusnigrofasciatus* appeared only at L5 (R2, 1,776 m). This distribution may relate to the size of the streams at the higher levels, which were generally smaller and with weaker currents, so pools were more common, which appeared to be a suitable habitat for dytiscids; most collecting of dytiscids was at depositional zones of the stream. This agrees with a general preference of this family for lentic systems (Miller and Bergsten 2016; Benetti et al. 2018).

Dryopidae was the third family in species richness (3 spp.) and was present at lower elevations, with *Dryopsmexicanus* and *Helichussuturalis* at levels 1–3, and *Elmoparnuspandus* only at level 1. This is a mostly tropical family, which appears to explain their presence at low elevations, although there are records at higher elevation in other areas (Huanachin-Quispe and Huamantico-Araujo 2018). This family includes species that may be observed in both lotic and lentic environments, however, many of the species may be present near the water margin or even outside (Jäch and Balke 2008), also their larvae are terrestrial. This particular biology may indirectly restrict the presence of adult dryopids at such lower elevation sites. During collecting, specimens were only found submerged associated to substrates.

The rest of the families were represented by one species each. Hydraenidae (*Hydraena* sp.) was observed at the five sampling levels, which agrees with the broad distribution of the group and that species of this genus occupy different types of habitats, from small streams to large rivers (Trizzino et al. 2013). Noteridae (*Notomicrussharpi*) was only present at level 4 (river 2, 1,619 m), which is above the previous known altitudinal record; as dytiscids, noterids prefer environments with slow current and some depth (Megna and Deler 2006), which includes the small pond (with macrophytes) at one side of the main stream where the only specimen was captured. Epimetopidae (*Epimetopusthermarum*), Gyrinidae (*Gyretesboucardi*), and Lutrochidae (*Lutrochus* sp.) were only recorded at level 1 (693 m). It is known that *Epimetopus* is attracted to lights (Perkins 2012), this agrees with our findings as specimens were captured with a bucket light trap. *G.boucardi* was collected in October, agreeing with White and Roughley’s (2008) time of emergence of late summer and early fall for the species; specimens were captured in an adjacent pool forming a large aggregation, *Lutrochus* sp. was only found at level 1, with specimens captured on macrophytes; this group is typically from lotic systems; however, it has been little studied in Mexico. Finally, Hydrophilidae (*Tropisternusfuscitarsis*) was only recorded at level 2; it is interesting this representative family was only present with one species, which was collected with bucket light trap, probably indicating a not very suitable habitat for the group in a volcanic-based ecosystem.

Species observed in levels 1–3 are usually of Neotropical affinity, while in levels 4 and 5 species with both Nearctic and Neotropical distribution increase. In general, most of the species are of Neotropical distribution with an affinity for the Pacific and Mesoamerican domains, which coincides with Morrone and Márquez (2001), who observed that the Coleoptera fauna of the Chiapas Highland province is related to the Veracruzan and Pacific Lowlands provinces, which are part of the Mesoamerican domain. The relationship between the Chiapas Highland province and Veracruzan and Pacific Lowlands provinces was confirmed by Morrone (2019). This general partition in two groups of altitudinal levels, 1–3 and 4 + 5, is supported by a PAE analysis, pointing out to a preliminary general pattern of altitudinal distribution for the aquatic beetle fauna of Volcán Tacaná.

## ﻿Conclusions

The aquatic beetle fauna of Volcán Tacaná presents a high diversity, with Elmidae, Dystiscidae, and Dryopidae as the most species-rich families, being responsible for 85% of the species. Some families (e.g., Hydraenidae and Elmidae) are distributed along all the altitudinal range, while Dytiscidae is present particularly at the higher altitudinal levels (1,619–1776 m); Noteridae is also present at high altitude, but only in a river located at 1,741 m. Remaining families, Dryopidae, Epimetopidae, Gyrinidae, Hydrophilidae, and Lutrochidae are present in lowlands (670–1,214 m). The aquatic beetle fauna of Volcán Tacaná presents a general partition in two well-defined groups: a lower altitude fauna (between 670, 934 and 1,150–1,214 m, levels 1–3) and a higher altitude fauna (between 1,619 and 1,776 m, levels 4 and 5). This fauna has an affinity to the Pacific and Mesoamerican biogeographic domains.

## Supplementary Material

XML Treatment for Dryops

XML Treatment for Elmoparnus

XML Treatment for Elmoparnus pandus

XML Treatment for Helichus

XML Treatment for Helichus suturalis

XML Treatment for Bidessonotus

XML Treatment for Bidessonotus championi

XML Treatment for Clarkhydrus

XML Treatment for Clarkhydrus

XML Treatment for Copelatus

XML Treatment for Copelatus distinctus

XML Treatment for Ilybiosoma

XML Treatment for Ilybiosoma flohrianum

XML Treatment for Laccophilus

XML Treatment for Laccophilus proximus

XML Treatment for Liodessus

XML Treatment for Liodessus affinis

XML Treatment for Neoclypeodytes

XML Treatment for Neoclypeodytes fryii

XML Treatment for Platambus

XML Treatment for Platambus americanus

XML Treatment for Rhantus

XML Treatment for Rhantus gutticollis

XML Treatment for Thermonectus

XML Treatment for Thermonectus nigrofasciatus

XML Treatment for Uvarus

XML Treatment for Uvarus subornatus

XML Treatment for Austrolimnius

XML Treatment for Austrolimnius formosus

XML Treatment for Austrolimnius sulcicollis

XML Treatment for Cylloepus

XML Treatment for Cylloepus atys

XML Treatment for Heterelmis

XML Treatment for Heterelmis glabra

XML Treatment for Heterelmis obesa

XML Treatment for Heterelmis obscura

XML Treatment for Heterelmis simplex

XML Treatment for Hexacylloepus

XML Treatment for Hexacylloepus metapa

XML Treatment for Hexanchorus

XML Treatment for Hexanchorus usitatus

XML Treatment for Huleechius

XML Treatment for Huleechius spinipes

XML Treatment for Macrelmis

XML Treatment for Macrelmis graniger

XML Treatment for Macrelmis leonilae

XML Treatment for Macrelmis

XML Treatment for Microcylloepus

XML Treatment for Microcylloepus inaequalis

XML Treatment for Microcylloepus troilus

XML Treatment for Microcylloepus

XML Treatment for Neoelmis

XML Treatment for Neoelmis apicallis

XML Treatment for Onychelmis

XML Treatment for Onychelmis longicollis

XML Treatment for Phanocerus

XML Treatment for Phanocerus clavicornis

XML Treatment for Xenelmis

XML Treatment for Xenelmis bufo

XML Treatment for Epimetopus

XML Treatment for Epimetopus thermarum

XML Treatment for Gyretes

XML Treatment for Gyretes boucardi

XML Treatment for Hydraena

XML Treatment for Hydraena

XML Treatment for Tropisternus

XML Treatment for Tropisternus fuscitarsis

XML Treatment for Lutrochus

XML Treatment for Lutrochus

XML Treatment for Notomicrus

XML Treatment for Notomicrus sharpi

## ﻿Appendix 1

**Table A1.** Collecting data of examined material of the new records of species for Mexico; all specimens are deposited at Colección Nacional de Insectos (CNIN), UNAM. LV1-LV5 = sampled levels; R1 and R2 = sampled rivers; m = male, f = female; * = specimens collected with bucket light trap (as explained in materials and methods).

Sample data (day/month/year)	ELMIDAE				DYTISCIDAE
	*Cylloepusatys* Hinton, 1946	*Hexacylloepusmetapa* Silva-Polizei, Barclay & Bispo, 2020	*Hexanchorususitatus* Spangler & Santiago-Fragoso, 1992	*Onychlemislongicollis* (Sharp, 1882)	*Bidessonotuschampioni* J. Balfour-Browne, 1947
**10/02/2018**	LV1R1 (2m)	LV1R1 (7m, 4f)			
**11/02/2018**	LV2R1 (1m)	LV2R1 (2f)			
**13/02/2018**				LV1R1 (1f)	
**17/02/2018**					LV4R2 (1m)
**10/03/2018**		LV1R1 (2m, 3f)	LV1R1 (10m, 8f); LV2R1 (1m, 2f)*		
**09/04/2018**			LV1R1 (10m, 14f)		
**10/04/2018**		LV2R1 (1m, 3f)			
**11/04/2018**				LV3R1 (1m); LV3R2 (2f)	
**13/04/2018**		LV3R2 (2m, 2f)			
**20/04/2018**	LV5R1 (1m, 1f)				
**07/05/2018**		LV1R1 (3m)	LV1R1 (44m, 58f); (59m, 76f)*		
**09/05/2018**	LV2R1 (1m, 1f)	LV2R1 (2m, 2f)		LV2R (1f)	
**11/05/2018**	LV3R1 (1f)	LV3R1 (1m, 2f)			
**12/05/2018**		LV3R2 (2m, 1f)	LV3R2 (28m, 26f); (2f)*		
**08/06/2018**	LV1R1 (1f)	LV1R1 (2m, 6f)			
**09/06/2018**		LV2R1 (4m, 9f)			
**15/06/2018**	LV4R2 (1m, 3f)				
**22/06/2018**	LV5 R1 (1m)				
**08/07/2018**		LV1R1 (3m, 5f)	LV1R1 (34m, 38f); (16m, 63f)*		
**09/07/2018**	LV1R1 (3m, 7f)	LV2R1 (45m, 31f)	LV2R1 (10m, 18f); (15m, 11f)*		
**10/07/2018**		LV3R2 (12m, 25f)	LV3R2 (17m, 24f)		
**11/07/2018**		LV3R1 (6m, 3f)	LV3R1 (32m, 25f)		
**13/07/2018**		LV4R2 (1m)			
**14/07/2018**	LV4R1 (1m); LV4R2 (1m)	LV4R2 (1f)			
**20/07/2018**	LV5R1 (1f)	LV5R1 (2m, 3f)			
**07/08/2018**	LV1R1 (2m, 1f)	LV1R1 (10m, 19f)	LV1R1 (1m); (1m, 2f)*		
**08/08/2018**	LV2R1 (1f)	LV2R1 (27m, 20f)			
**10/08/2018**	LV3R1 (1m)	LV3R1 (8m, 13f); LV3R2 (20m, 43f)	LV3R1 (1m)		
**12/08/2018**	LV4R2 (3m, 4f); LV4R2 (3m, 4f)				
**13/08/2018**		LV4R2 (1f)			
**17/08/2018**	LV5R2 (6m, 2f)				
**04/09/2018**		LV1R1 (10m, 3f)	LV1R1 (34m, 17f)		
**05/09/2018**		LV2R1 (1m)			
**07/09/2018**		LV3R1 (4m, 2f)			
**03/10/18**		LV1R1 (2m, 2f)	LV1R1 (11m, 26f)		
**04/10/18**			LV2R1 (6m, 13f)		
**12/10/2018**	LVR2 (2f); LV5R2 (1m, 1f)				
**02/11/2018**			LV1R1 (5m, 6f); LV2R1 (10m, 5f)		
**07/11/2018**		LV3R2 (1m, 2f)	LV3R2 (4m, 8f)		
**23/11/2018**	LV5R2 (2m)	LV5R1 (1f)			
**03/12/2018**		LV2R1 (2m, 3f)	LV2R1 (3m, 7f)		
**04/12/2018**	LV4R2 (2f)				
**06/12/2018**			LV3R2 (3m, 13f)		
**09/12/2018**		LV5R1 (2m)			
**04/01/2019**	LV2R1 (2f)		LV2R1 (7m, 15f)		
**06/01/2019**		LV3R2 (1m, 2f)	LV3R1 (2m, 2f)		
**04/02/2019**	LV1R1 (4f)	LV2R1 (1m, 3f)	LV1R1 (1m, 7f); LV2R1 (6m, 10f)		
**05/02/2019**			LV3R1 (12m, 19f)		
